# No association between apolipoprotein E or N‐Acetyltransferase 2 gene polymorphisms and age‐related hearing loss

**DOI:** 10.1002/lary.24898

**Published:** 2014-08-22

**Authors:** Piers Dawes, Hazel Platt, Michael Horan, William Ollier, Kevin Munro, Neil Pendleton, Antony Payton

**Affiliations:** ^1^ School of Psychological SciencesSalford Royal NHS HospitalManchesterUnited Kingdom; ^2^ Centre for Integrated Genomic Medical ResearchThe University of Manchester, Salford Royal NHS HospitalManchesterUnited Kingdom; ^3^ Central Manchester University Hospitals NHS Foundation TrustManchester Academic Health Science Centre, Salford Royal NHS HospitalManchesterUnited Kingdom; ^4^ Salford Research and DevelopmentSalford Royal NHS HospitalManchesterUnited Kingdom; ^5^ Clinical GerontologySalford Royal NHS HospitalManchesterUnited Kingdom

**Keywords:** Age‐related hearing loss, presbyacusis, APOE, N‐acetyltransferase 2

## Abstract

**Objectives/Hypothesis:**

Age‐related hearing loss has a genetic component, but there have been limited genetic studies in this field. Both N‐acetyltransferase 2 and apolipoprotein E genes have previously been associated. However, these studies have either used small sample sizes, examined a limited number of polymorphisms, or have produced conflicting results. Here we use a haplotype tagging approach to determine association with age‐related hearing loss and investigate epistasis between these two genes.

**Study Design:**

Candidate gene association study of a continuous phenotype.

**Methods:**

We investigated haplotype tagging single nucleotide polymorphisms in the N‐acetyltransferase 2 gene and the presence/absence of the apolipoprotein E ε4 allele for association with age‐related hearing loss in a cohort of 265 Caucasian elderly volunteers from Greater Manchester, United Kingdom. Hearing phenotypes were generated using principal component analysis of the hearing threshold levels for the better ear (severity, slope, and concavity). Genotype data for the N‐acetyltransferase 2 gene was obtained from existing genome‐wide association study data from the Illumina 610‐Quadv1 chip. Apolipoprotein E genotyping was performed using Sequenom technology. Linear regression analysis was performed using Plink and Stata software.

**Results:**

No significant associations (*P* value, > 0.05) were observed between the N‐acetyltransferase 2 or apolipoprotein E gene polymorphisms and any hearing factor. No significant association was observed for epistasis analysis of apolipoprotein E ε4 and the N‐acetyltransferase 2 single nucleotide polymorphism rs1799930 (NAT2*6A).

**Conclusion:**

We found no evidence to support that either N‐acetyltransferase 2 or apolipoprotein E gene polymorphisms are associated with age‐related hearing loss in a cohort of 265 elderly volunteers.

**Level of Evidence:**

N/A. *Laryngoscope*, 125:E33–E38, 2015

## INTRODUCTION

Age‐related hearing loss (ARHL) (also known as presbyacusis) is common. Population‐based studies have reported a prevalence of hearing impairment of up to 45.9% in adults aged over 40 years.^1^, ^2^ Hearing loss has a substantial impact on quality of life via impaired communication and is associated with social isolation, depression, reduced physical well‐being, cognitive decline, unemployment, and low‐grade jobs.^3^, ^4^ An international report concluded that hearing impairments cost Europe 213 billion Euros per year.^5^ With an aging society, the proportion of those with hearing loss is increasing, and hearing loss will be in the top 10 disease burdens in high‐ and middle‐income countries by 2030.^6^

Heritability studies of ARHL in humans have estimated that 25% to 75% of the variance in ARHL has a genetic component, depending on the definition and measurement of ARHL and the population in question.^7^, ^8^, ^9^, ^10^ Both apolipoprotein E (APOE) and N‐acetyltransferase 2 (NAT2) genes have been associated with ARHL. NAT2 codes for an enzyme that metabolizes carcinogens, including hydrazine and arylamine drugs. Genetic polymorphisms within the NAT2 gene are associated with the rate of catalytic activity; therefore, they are predisposed toward drug toxicity.^11^ Three independent studies have reported a significant association between a NAT2 single nucleotide polymorphism (SNP) (NAT2*6A; rs1799930) and ARHL.^12^, ^13^, ^14^ The original study by Unal et al. investigated four NAT2 polymorphisms using a cohort of 68 ARHL cases and 98 controls (Turkish‐Caucasian) and reported a significant association with SNP rs1799930 and ARHL.^12^ An attempt at replication was later performed by Van Eyken et al., who found a significant association in a large cohort of general Europeans (n = 1695) but not in a Finnish cohort (n = 514).^13^ Finally, Bared et al. successfully replicated the association using 55 cases and 79 controls (a mix of mainly Hispanic and non‐Hispanic subjects).^14^ A more recent study of 55 presbycusis subjects found no association between NAT2 polymorphisms and the shape of their audiometric patterns.^15^

APOE is a gene strongly associated with the neurodegenerative condition, Alzheimer's disease—with the APOE ε4 allele predisposing to susceptibility.^16^, ^17^ In contrast to the association with Alzheimer's disease, it has been reported that the APOE ε4 allele was significantly less common in the study population with hearing loss (n = 89) compared to the frequency reported in the general population, implying a protective effect.^18^ Interestingly, APOE has been shown to up‐regulate N‐acetyltransferase expression, suggesting that these genes may exert an epistasis effect on ARHL.^19^

The aim of this study was to attempt replication of the reported associations between ARHL and APOE ε4 and NAT2 genes and to investigate a possible gene–gene interaction in a cohort of 265 elderly, community‐dwelling Caucasian volunteers from the United Kingdom using a haplotype tagging SNP approach.

## MATERIALS AND METHODS

### Subjects

Participants were a subset of the University of Manchester Longitudinal Study of Cognition cohort, which comprised 265 Caucasian residents (145 female) of Greater Manchester, United Kingdom.^20^ The average age of participants was 72 years (range 59–88 years) at the time of hearing assessment and genetic sampling, which was collected between the years 1998 and 2000. Participants provided written informed consent for collection of a DNA sample from venous blood, and the study was approved by the University of Manchester Research Ethics Committee. Hearing sensitivity was assessed using pure tone audiometry between 1997 and 1999. Pure‐tone hearing threshold levels (over 0.25–8 kHz) for each ear were measured using standard audiometric techniques described by the British Society of Audiology.^21^

### Description of the Phenotype

**Figure 1:**
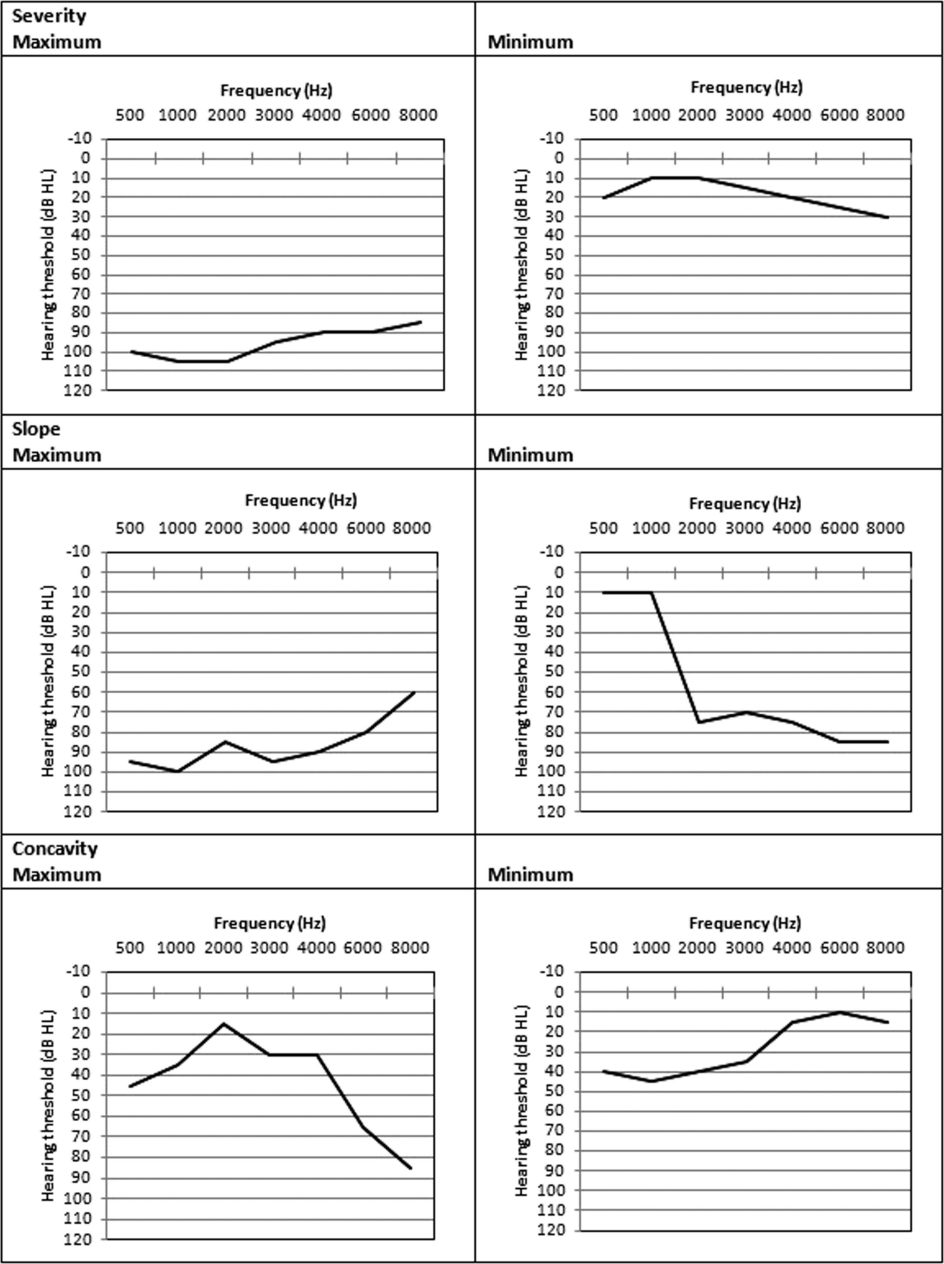
Summary of PC analysis showing the three PCs (minimum and maximum) representing severity (PC1), slope (PC2), and concavity (PC3). PC = principle components. The three principle components (representing severity, slope, and concavity) capture 87.1% of the variance in hearing ability measured using the better ear. The mean hearing threshold level in the better ear was 25 dB (SD = 12; range −3 to 91 dB, across 0.25, 0.5, 1, 2, 4, and 8 kHz). Hearing threshold levels increased with age and were poorer for men than for women, and so were adjusted for age and sex.

The phenotype was described following the method described by Huyghe et al. (2008) and Van Laer et al. (2011), which involved principal component analysis of the audiometric thresholds for the better ear.^22^, ^23^ The better ear was taken as the ear with the lowest (i.e., better) average threshold over 0.25 to 8 kHz. ARHL would be expected to be symmetrical across the ears; thus, using a better‐ear threshold minimizes the impact of idiopathic factors that may only affect one ear. The mean hearing threshold level in the better ear was 25 dB (SD = 12; range −3 to 91 dB, across 0.25, 0.5, 1, 2, 4, and 8 kHz). Hearing threshold levels increased with age and were poorer for men than for women, and so they were adjusted for age and sex. The effect of age was controlled by regressing log‐transformed thresholds [log_10_(20 + threshold)] for each measured frequency on age, age squared, and age cubed. To correct for sex, adjustment for age was made for males and females separately. The resulting residuals of each regression were then scaled and combined, with principal component analysis performed on the scaled residuals (Fig. 1). The first three components (Table 1 and Fig. 1) capture 87.1% of the variation and correspond to those described by Huyghe et al. (2009) and Van Laer et al. (2011).^22^, ^23^ Component 1 (PC1) is a “severity” variable, providing an overall index of hearing level. PC2 and PC3 are “shape” variables. PC2 contrasts low and high frequencies and is a measure of audiometric slope. PC3 contrasts middle frequencies with low and high frequencies, corresponding to the concavity of the audiogram. Huyghe et al. (2009) reported substantial heritability estimates for the three components, which were highest for PC1 (66.3%) and lowest for PC2 (27.2%).^22^

**Table 1:** Eigenvalue Coefficients of the First Three Principal Components.

Trait	PC1	PC2	PC3
Eigenvalue	4.34	1.80	0.82
% of variance	54.30	22.49	10.30
0.25 kHz	0.50	0.75	0.24
0.5 kHz	0.62	0.73	0.11
1 kHz	0.75	0.45	−0.13
2 kHz	0.80	−0.15	−0.45
3 kHz	0.84	−0.18	−0.32
4 kHz	0.85	−0.35	−0.09
6 kHz	0.80	−0.40	0.31
8 kHz	0.66	−0.40	0.57

Component 1 (PC1) is a “severity” variable, providing an overall index of hearing ability. PC2 and PC3 are “shape” variables. PC2 contrasts low and high frequencies and is a measure of audiometric slope. PC3 contrasts middle frequencies with low and high frequencies, corresponding to the concavity of the audiogram.

PC = principle components.

### Power Calculation

Our power calculation was based on the original study by Unal et al., who reported association using 68 cases and 98 controls. This sample size has 85% power to detect a genotype relative risk of 1.7 (genetic risk of approximately 10%) assuming an additive model, significance 0.05 (two‐tailed), and minor allele frequencies (MAF) of 0.31 (MAF of NAT2*6A; rs1799930). Our sample size of 265 had over 99% power to replicate this association assuming the same parameters. For APOE (MAF of APOEε4, 0.13) we had 80% power to detect a genetic effect size of 3% (significance 0.05, two‐tailed) assuming an additive model for a continuous trait.

### Genotyping and Quality Control of the Data

Genome‐wide Association Study data were available on all volunteers. Genotyping was performed using the Illumina 610‐Quadv1 chip (Illumina, Inc., San Diego, CA). Individuals were excluded from further analysis if there was a disagreement between genetic and reported gender. Relatedness between participants was investigated, and one individual was removed for any related pair of individuals. Samples showing evidence of non‐Caucasian descent by multidimensional scaling were also removed. All 265 volunteers had both genotype and phenotype data, with the exception of NAT2 SNPs rs1495750, rs7013253, and rs1565684, for which successful genotyping data was available on 261, 264, and 263 volunteers, respectively. APOE genotyping was performed using Sequenom technology (Sequonom Inc, San Diego, CA) using the iPLEX method. This method has been described previously by Ghebranious et al. (2005).^24^

### Selection and Analysis of Haplotyping Tagging SNPs

SNPs were selected from those genotyped on the Illumina chip that flanked the NAT2 gene, including 15k base pairs on either side of the gene to cover potential regulatory regions. A total of 13 haplotype tagging SNPs (htSNPs) were selected using the Tagger program in Haploview version 4.2 (Broad Institute, Cambridge, MA).^25^ The htSNPs included NAT2*6A (rs1799930), which was the SNP reported to be associated with ARHL by the previous studies described above. APOE was analyzed for the presence or absence of the ε4 allele. SNP linear regression analysis, epistasis analysis, Hardy‐Weinberg Equilibrium (HWE), and calculation of MAF were performed in Plink version 1.07 (http://pngu.mgh.harvard.edu/purcell/plink/).^26^ Analysis of APOE was performed in Stata 8 (StataCorp LP, College Station, TX).

## RESULTS

Table 2 summarizes the NAT2 htSNPs characteristics (base pair position on chromosome 8, MAF, HWE, and number of individuals successfully genotyped) and linear regression analysis *P* values. All SNPs were in HWE. No significant associations (*P* value, > 0.05) were observed between any of the NAT2 htSNPs and any hearing phenotype. Two SNPs showed a nonsignificant trend (rs13277723, *P* value, 0.06 and rs6998197, *P* value, 0.08) for PC3 and PC2, respectively, although these are likely the result of multiple tests. No correction for multiple testing was applied due to the initial results showing no significant association.

**Table 2:** Summary Statistics for NAT2 Haplotype Tagging SNPs Analyzed Against Hearing Phenotypes.

SNP	BP	MAF	HWE	Alleles	Number of Observations	PC1	PC2	PC3
						*P* Value	*P* Value	*P* Value
rs7006687	18277862	0.44	0.49	T:C	265	0.48	0.36	0.80
rs1495750	18282666	0.44	0.98	G:A	261	0.84	0.46	0.38
rs13277723	18285766	0.48	0.79	T:C	265	0.92	0.63	0.06
rs7013253	18287748	0.33	1	A:G	264	0.40	0.80	0.15
rs1565684	18290944	0.47	0.94	C:T	263	0.56	0.42	0.60
rs1799930^a^	18302383	0.31	1	G:A	265	0.75	0.48	0.86
rs1208	18302596	0.42	0.87	A:G	265	0.28	0.18	0.76
rs4646251	18305496	0.06	0.47	C:G	265	0.83	0.45	0.38
rs1495747	18307141	0.27	0.36	C:T	265	0.48	0.58	0.84
rs2410561	18311054	0.15	0.52	G:A	265	0.88	0.24	0.84
rs12545528	18316228	0.41	0.93	G:T	265	0.61	0.49	0.40
rs1495741	18317161	0.22	0.58	A:G	265	0.98	0.17	0.80
rs6998197	18318276	0.48	0.84	T:C	265	0.66	0.08	0.89

Base pair position (BP) on chromosome 8; Minor Allele Frequency (MAF); Hardy‐Weinberg Equilibrium (HWE); Base pair changes (Alleles), Total number genotyped and used in the analysis; P‐value of 3 phenotypes analysed (PC1–3).

a rs1799930 is NAT2*6A investigated in earlier studies.

HWE = Hardy‐Weinberg Equilibrium; MAF = minor allele frequencies; PC = principle components; SNP = single nucleotide polymorphism.

The frequency of the APOE ε4 allele was 13.1%, which is similar to reported frequencies in healthy Caucasian populations.^27^ Linear regression analysis of APOE also failed to find a significant association with the three hearing phenotypes, although there was a nonsignificant trend between PC1 (overall index of hearing loss severity) and the presence of the ε4 allele that was associated with reduced hearing ability (*P* value, 0.07; beta value, −0.94). No correction for multiple testing was applied to this value.

No significant association was observed for SNP × APOE allele epistasis analysis of APOEε4 and rs1799930 (*P* value set to detect significance ≥ 0.05).

## DISCUSSION

Hearing loss has traditionally been thought of as an inevitable consequence of aging. Although it is true that all individuals will experience some degree of hearing loss, there is a wide range of rates of progression and severity.^28^ A number of environmental risk factors for hearing loss have been identified, including noise exposure, tobacco, alcohol use, diet, cardiovascular disease, and use of ototoxic drugs.^29^, ^30^, ^31^, ^32^, ^33^, ^34^, ^35^, ^36^ Many of these can be adjusted, and thus offer avenues for prevention. Genetic factors may also increase risk for hearing loss and may interact with environmental factors, although research has been more limited in this area.^37^, ^38^

Here we investigated polymorphisms spanning two genes that have previously been associated with ARHL. The NAT2 gene product catalyses the acetylation of hydrides and amines in medicines and the carcinogenic compounds. A missense SNP within the NAT gene (NAT2*6A; rs1799930; G > A; Arg > Gln), which has been shown to influence the rate of acetylation (where the A allele is correlated with lower acetylation), has been associated with ARHL in three independent studies.^12^, ^13^, ^14^ These studies reported the association in Caucasian populations, although the Van Eyken study did not replicate the finding using a Finnish cohort. In contrast, we did not find a significant association between this SNP and ARHL.

Genetic differences caused by population stratification may account for the observed differences in results. Van Eyken used data collected from seven different European countries, which were combined into a large “general European population” (Belgium, UK, The Netherlands, Germany and Italy; n = 1695) and a Finnish population (n = 514).^13^ The SNP rs1799930 AA genotype frequency in our cohort was similar to that reported by Van Eyken for their “general Europeans” (9.8 and 9.0%; range 6%–10%), respectively) although the Finnish population had a slightly lower genotype frequency of 6%. It should also be noted that the Van Eyken study performed analysis on five SNPs from three genes, and their significance value (*P* value, 0.013) was uncorrected for multiple testing. The other two studies by Unal et al. and Bared et al. used a case‐control design. Unal et al. used 68 cases from a Turkish population, and Bared et al. investigated 55 cases from a mixed white Hispanic and white non‐Hispanic populations, with AA genotype frequencies for cases of 7.7% and 12% and for controls 4.1% and 0.0%, respectively.^12^, ^14^ This wide range of genotype frequencies may be attributed to population stratification, but this may be a consequence of small sample size in the case of the latter two studies. Once again, no correction was applied for multiple testing in the Unal et al. and Bared et al. studies, despite multiple polymorphisms being analyzed (uncorrected *P* value, 0.032 and 0.0086, respectively). Correction would have rendered all the studies mentioned above nonsignificant, although the consistency of the three significant associations (assuming no publication bias) adds support to their findings.

Between‐study variation also existed for the hearing loss phenotype measurements and may have contributed toward nonreplication. The Unal et al. and Bared et al. studies used a case‐control approach in which mean hearing level was greater than 30 dB for cases.^12^, ^14^ Both the Van Eyken study and our study analyzed volunteers using hearing phenotypes as a continuous trait, with Van Eyken taking two measurements at high and low frequency.^13^ The phenotype we used was that described by Huyghe et al. (2008) and Van Laer et al. (2011), which involved principal components analysis of the audiometric thresholds for the better ear, adjusting for age and sex.^22^, ^23^ The three principle components that were generated represented overall hearing loss severity, as well as slopes at high, medium, and low frequencies, which effectively matches all the phenotypes of the previous studies. A more recent study of ARHL that included the investigation of the rs1799930 and the audio profiles of 47 individuals (aged 50 or over, hearing loss > 25 dB) also observed no significant association.^15^

It has been hypothesized by O'Grady et al. that the ε4 allele of APOE may predispose individuals to ARHL by the same mechanisms as for Alzheimer's disease (via predisposition to auditory neuropathy) or atherosclerotic vascular disease (via ischemic injury to the cochlea).^18^ In contrast to this theory, the authors found that APOE ε4 was at a lower frequency in a study population with sensorineural hearing loss (89 subjects, median age 64, 38 females) compared to the general population, suggesting a protective influence. As with our NAT2 findings, we found no significant association between the ε4 allele and hearing loss, although we did see a nonsignificant trend (uncorrected *P* value, 0.07) that indicated the ε4 allele was increasing susceptibility to ARHL. In addition, we found no evidence of a gene–gene interaction between the NAT2 SNP rs1799930 and the presence or absence of the ε4 allele, despite a previous study finding that APOE regulates NAT2 expression.^19^

Inadequate statistical power has been an issue for association studies of complex genetic traits, which has resulted in a large number of false positive publications.^39^ Although our sample size of 265 would be considered small for reporting a new finding, the aim of our analysis was to replicate existing findings using a larger sample size than the original reports. To this end, our power calculation suggested that we had a greater power than the initial studies with which to test the robustness of the original associations. However, we cannot exclude the possibility that the polymorphisms are exerting a smaller effect size than our current sample size allows us to detect. Indeed, in complex genetic diseases and conditions, reported effect sizes of less than 1% are common.^40^ Our results highlight the need for the use of adequate power and for the use of an independent replication cohort when a gene is first implicated with a complex genetic condition such as ARHL.

Given the high prevalence, the substantial burden of hearing loss, and the limited effectiveness and under‐use of current treatments, identification of new and more effective treatments—as well as the prevention of hearing loss—is a research priority.^3^ Identifying the genetic basis for ARHL will provide new targets for drug development. Unfortunately, current genetic studies have tended to provide conflicting results.

## CONCLUSION

We found no evidence that the NAT2 and APOE genes are involved in ARHL using a replication population of 265 elderly volunteers. Our work highlights the current limitations of previous studies that have investigated the genetics of ARHL, and we recommend that future study designs use increased sample sizes, better defined phenotypes, and longitudinal measurements.
